# Comparison of airway pressures and expired gas washout for nasal high flow versus CPAP in child airway replicas

**DOI:** 10.1186/s12931-021-01880-z

**Published:** 2021-11-10

**Authors:** Kelvin Duong, Michelle Noga, Joanna E. MacLean, Warren H. Finlay, Andrew R. Martin

**Affiliations:** 1 Department of Mechanical Engineering, University of Alberta, Edmonton, Canada; 2 Department of Radiology and Diagnostic Imaging, University of Alberta, Edmonton, Canada; 3 Department of Pediatrics and Women & Children’s Health Research Institute, Faculty of Medicine & Dentistry, University of Alberta, Edmonton, Canada; 4 Stollery Children’s Hospital, Edmonton, Canada; 5 10-324 Innovation Centre for Engineering, University of Alberta, Edmonton, AB T6G 1H9 Canada

**Keywords:** Obstructive sleep apnea, Continuous positive airway pressure, Nasal high flow, Nasal cannula, Adherence, Tracheal pressure, End-tidal carbon dioxide

## Abstract

**Background:**

For children and adults, the standard treatment for obstructive sleep apnea is the delivery of continuous positive airway pressure (CPAP). Though effective, CPAP masks can be uncomfortable to patients, contributing to adherence concerns. Recently, nasal high flow (NHF) therapy has been investigated as an alternative, especially in CPAP-intolerant children. The present study aimed to compare and contrast the positive airway pressures and expired gas washout generated by NHF versus CPAP in child nasal airway replicas.

**Methods:**

NHF therapy was investigated at a flow rate of 20 L/min and compared to CPAP at 5 cmH_2_O and 10 cmH_2_O for 10 nasal airway replicas, built from computed tomography scans of children aged 4–8 years. NHF was delivered with three different high flow nasal cannula models provided by the same manufacturer, and CPAP was delivered with a sealed nasal mask. Tidal breathing through each replica was imposed using a lung simulator, and airway pressure at the trachea was recorded over time. For expired gas washout measurements, carbon dioxide was injected at the lung simulator, and end-tidal carbon dioxide (EtCO_2_) was measured at the trachea. Changes in EtCO_2_ compared to baseline values (no intervention) were assessed.

**Results:**

NHF therapy generated an average positive end-expiratory pressure (PEEP) of 5.17 ± 2.09 cmH_2_O (mean ± SD, n = 10), similar to PEEP of 4.95 ± 0.03 cmH_2_O generated by nominally 5 cmH_2_O CPAP. Variation in tracheal pressure was higher between airway replicas for NHF compared to CPAP. EtCO_2_ decreased from baseline during administration of NHF, whereas it increased during CPAP. No statistical difference in tracheal pressure nor EtCO_2_ was found between the three high flow nasal cannulas.

**Conclusion:**

In child airway replicas, NHF at 20 L/min generated average PEEP similar to CPAP at 5 cm H_2_O. Variation in tracheal pressure was higher between airway replicas for NHF than for CPAP. The delivery of NHF yielded expired gas washout, whereas CPAP impeded expired gas washout due to the increased dead space of the sealed mask.

**Supplementary Information:**

The online version contains supplementary material available at 10.1186/s12931-021-01880-z.

## Background

Obstructive sleep apnea (OSA) is a common sleep-related breathing disorder in which an individual’s upper airway is obstructed, causing partial to complete interruptions in their breathing. OSA affects both adults and children, but the consequences of the disorder may differ between the two groups. The negative impacts of OSA on cognitive, learning, and behavioural functions are more serious in children than in adults [1–3]. Other complications in children include cardiovascular complications and impacts on growth [1, 2, 4, 5]. OSA is estimated to affect between 1 and 10% of children [1, 6–8].

The delivery of continuous positive airway pressure (CPAP) is an effective treatment for OSA in children [9, 10]. CPAP restores breathing and sleep by acting as a pneumatic stent to prevent the collapse of the upper airways. Typically, a nasal/facial mask, preferably selected to conform as best as possible to the individual’s facial geometry, is used to administer CPAP [11]. Though effective, adherence to the therapy is poor due to discomfort [12, 13]. Multiple factors contribute to discomfort, such as mask leak, skin irritation, and/or pressure sores [14, 15]. With the goal of improving adherence to CPAP therapy, several groups have investigated improvements to the comfort of the mask interface [16–19]. However, other groups have explored alternative forms of non-invasive respiratory support, including administration of nasal high flow (NHF) therapy [20, 21].

**Fig. 1:**
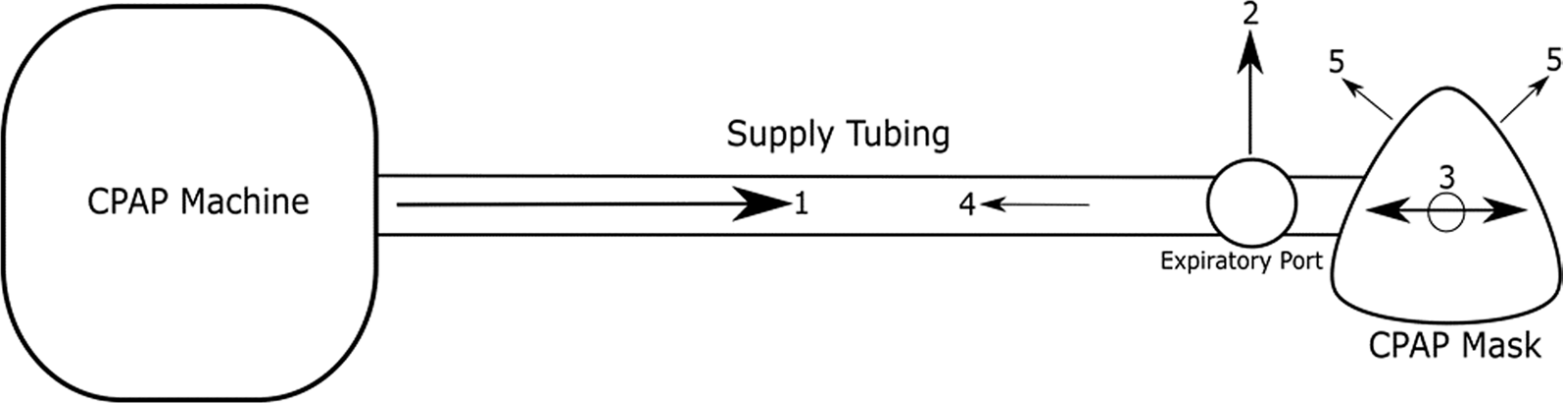
Schematic of CPAP therapy with arrows indicating the flow direction of air. (Arrow 1) Flow of gas (air or air/oxygen mixture) provided by the CPAP machine. (Arrow 2) Flow of gas that exits the expiratory port on the supply tube. (Arrow 3) Cyclic flow of gas from the patient during inspiration and expiration. (Arrow 4) Backflow of air that may occur during expiration at high flow rate. (Arrow 5) Flow of air out of the mask when leaks exist between the mask cushion and face. *CPAP* continuous positive airway pressure

**Fig. 2:**
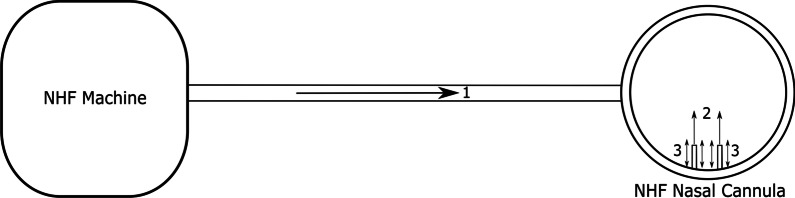
Schematic of nasal high flow therapy with arrows indicating the flow direction of delivered gas (air or air/oxygen mixture). (Arrow 1) Flow of gas provided by the NHF machine (constant). (Arrow 2) Flow of gas that exits the nasal cannula prongs into the patient’s nostrils. (Arrow 3) Cyclic flow of gas entrained by the patient during inspiration, or expelled during expiration, occurring around the nasal cannula prongs. *NHF* nasal high flow

The most obvious difference in the administration of CPAP versus NHF is in the interface used. For CPAP, breathing gas is typically delivered to the patient through a tightly-fitted nasal or facial mask. Air, or an air/oxygen mixture, is delivered from a CPAP machine to the mask through a supply tube with an expiratory port (Fig. 1). In contrast, during NHF therapy, air, or an air/oxygen mixture, is delivered through an open interface: a high flow nasal cannula. Unlike CPAP, no expiratory port is included in the supply tube, as exhaled gases are vented to the room through the open space around the nasal cannula prongs (Fig. 2). For CPAP, the expiratory port acts both as an outlet for expired air, as well as a means through which the CPAP machine generates pressure in the supply tube and mask. During breathing, the CPAP machine monitors pressure and continuously adjusts the flow rate of gas it delivers, in order to maintain a constant pressure in the supply tubing and mask. In contrast, during NHF therapy, gas is supplied at a constant flow rate, which does not adjust according to patient breathing. Pressure is not monitored during NHF therapy.

The delivery of NHF for OSA in children has been investigated as an alternative to mask-based CPAP [20–22]. NHF therapy generates positive airway pressure through the delivery of humidified air or air/oxygen mixtures at high flow rates through nasal cannulas. In studies by Hawkins et al. [22] and Amaddeo et al. [21], both groups assessed NHF therapy in children who were intolerant to CPAP therapy. NHF therapy was shown to have good compliance in children and was able to reduce respiratory events [21, 22]. The open interface of the nasal cannula may be more comfortable and tolerable than CPAP masks for overnight use [20–22]. Furthermore, in children, CPAP has been associated with hindered development of the face due to use of tight-fitting masks [23]. The use of NHF may avoid this issue. In addition to positive airway pressure, NHF therapy is known to provide washout of the nasopharyngeal dead space [24]. Washout may improve gas exchange, potentially contributing to correction of hypopneas and apneas in children with OSA [21, 22]. These benefits make NHF therapy a promising alternative for CPAP-intolerant children.

In the present work, upper airway pressures and carbon dioxide washout were compared between NHF and CPAP therapy in vitro using child airway replicas coupled to a lung simulator.

## Methods

In this in vitro study, the delivery of NHF through nasal cannula was compared with the delivery of CPAP through a nasal mask. The study was conducted using the upper airway replicas of 10 child subjects, with two main comparative measurements: tracheal pressures and end-tidal carbon dioxide concentration (EtCO_2_). Tracheal pressures were separated into four parameters: positive end-expiratory pressure (PEEP), peak expiratory pressure (PEP), minimum inspiratory pressure (MIP), and average inspiratory pressure (AIP).

### Child airway replicas

The 10 upper airway replicas, which include the nose-throat airway and terminate at the trachea, were previously fabricated in our research group based on computed tomography (CT) scan data of 10 child subjects, between the ages of 4 and 8 years, as reported by Paxman et al. [25]. All subjects had been previously scanned for indications other than airway pathology and the airway was confirmed to be normal prior to inclusion of data. The replicas were 3D printed (Objet Eden 350V; Stratasys Ltd., MN, USA) using a rigid opaque photopolymer material (VeroGray; Stratasys Lt., MN, USA). Further details on the fabrication of the replicas can be found in the work by Paxman et al. [25]. For the present study, branching airways downstream of the carina were removed from the replicas, and 3D printed adapters were attached to the exit of each replica to standard 22 mm breathing circuit tubing. Demographic data and geometric properties of the replicas are presented in Table 1.

**Table 1:** Demographic and geometric data for airway replicas used in the present study

Subject number	Age	Sex	Height (m)	Weight (kg)	Airway volume (mL)	Area of nostrils (mm^2^)
2	5	M	1.17	22.9	40.4	55
3	5	M	1.12	20.0	35.1	115
5	6	F	1.12	18.0	19.1	85
6	6	F	1.18	21.5	32.1	66
9	5	M	1.13	20.0	21.0	80
10	4	F	0.99	16.0	19.2	58
11	8	M	1.25	24.5	48.4	100
12	6	F	1.24	24.0	22.2	86
13	7	F	1.21	20.0	32.5	84
14	4	F	1.00	16.0	18.6	56

### Experimental apparatus

A lung simulator (ASL 5000 Breathing Simulator; IngMar Medical, Pittsburgh, PA, USA) was used to simulate tidal breathing through the replicas.

For the present study, breathing frequency (f) and inspiratory/expiratory (i/e) ratio were fixed at 17 breaths per minute (BPM) and 0.85, respectively. Tidal volume (V_t_) was fixed at 10 mL/kg body weight yielding a range of 160–245 mL. These breathing parameters were selected as typical in studies involving high flow and CPAP delivery to children in this age group [26–28]. With these three parameters, the inspiratory and expiratory phases of a breath were modeled as half-sine waves with no inspiratory or expiratory pause.

**Fig. 3:**
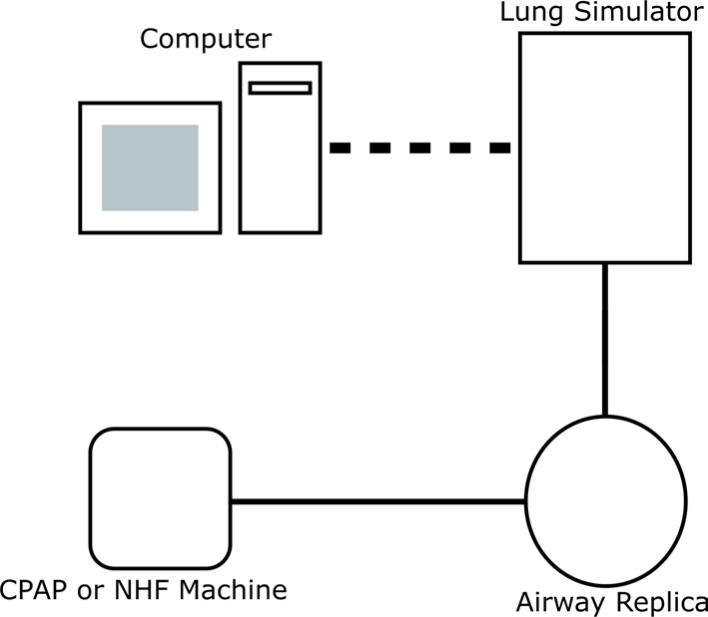
Schematic of experimental apparatus for measuring tracheal pressures

For tracheal pressures, the intervention, either CPAP or NHF, was applied to the replica which was connected to the lung simulator through standard 22 mm breathing circuit tubing (Fig. 3). The length of tubing was kept short to minimize pressure losses and measured 17.0 cm.

**Fig. 4:**
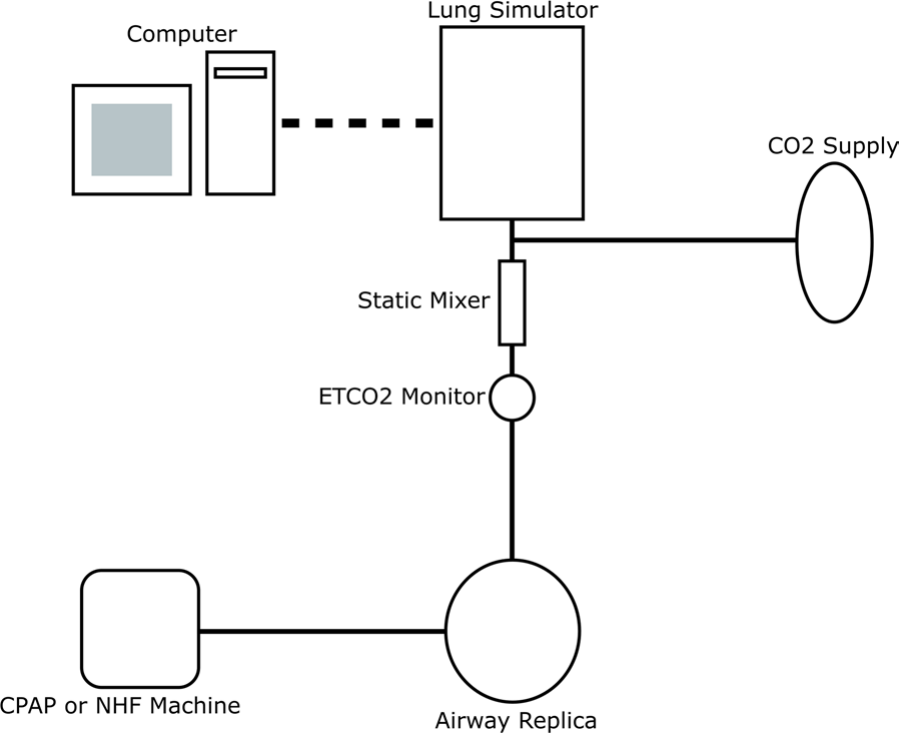
Schematic of experimental apparatus for measuring EtCO_2_

For EtCO_2_, an intervention was applied to the replica, which was connected to the lung simulator through two airway adapters and a static mixer (Fig. 4). A capnograph (EMMA Capnograph; Masimo, Irvine, CA) was attached to the adult/pediatric EMMA Airway Adapter (Masimo, Irvine, CA), positioned between the replica and mixer, to measure EtCO_2_ through infrared spectroscopy. The resulting EtCO_2_ was displayed as a running average on the screen of the capnograph in mmHg along with the respiratory rate. A straight connector with 7.6 mm port (1964000; Intersurgical, Wokingham, Berkshire, UK) was positioned between the mixer and lung simulator, and used for injection of CO_2_. The mixer was used to ensure that the supplied CO_2_ was well mixed in the breathing circuit before reaching the capnograph [29]. The internal volume of the connection between the replica and the lung simulator measured 59.2 mL.

A constant flow of 100% CO_2_ was bled inline to achieve 5% EtCO_2_ as a baseline during simulated breathing through each replica without any intervention applied. EtCO_2_ was converted from mmHg to % CO_2_ at an average atmospheric pressure of 707.32 mmHg (Edmonton, Alberta, Canada) over the testing period of the experiments. The required CO_2_ injection rates ranged from 60 to 130 mL/min depending on the replica, and are displayed in Table 2. EtCO_2_ values measured during each tested intervention were reported as a change in % CO_2_ from baseline.

**Table 2:** Tidal volume and CO_2_ injection rates for each airway replica

Subject number	Tidal volume (mL)	CO_2_ injection rate (mL/min)
2	229	115
3	200	95
5	180	80
6	215	100
9	200	95
10	160	60
11	245	130
12	240	130
13	200	85
14	160	60

### Nasal high flow

NHF was delivered with a humidified Nasal High Flow system, Airvo 2, which was provided by Fisher & Paykel Healthcare (Auckland, New Zealand). During the study, the supplied flow was set at a flow rate of 20 L/min, consistent with the flow rate used in studies by McGinley et al. and Amaddeo et al. that investigated NHF for treating OSA in children with a similar age range as the present study [20, 21]. Temperature was set at 34 °C with supplied oxygen concentration set at 21%. Three high flow nasal cannulas were tested, which were provided by Fisher & Paykel Healthcare: the Optiflow 3S Nasal Cannula (small, OPT1042), the Optiflow + Nasal Cannula (small, OPT942), and the Optiflow Junior 2 Nasal Interface (XL, OJR418). The inner and outer diameters for each nasal cannula prong are provided in Table 3.

**Table 3:** Inner and outer diameters of nasal cannula prongs

Nasal cannula	Diameter (mm)	
	Inner	Outer
Optiflow 3S	4.2	5.0
Optiflow +	4.1	4.9
Optiflow Junior 2	3.0	3.8

During administration of NHF, PEEP is generated in the upper airway as supplied flow from the cannula reverses direction and exits the airway around the obstruction created by the presence of the nasal prongs positioned in the nares. In fluid mechanics, pressure losses due to obstructions are commonly modeled as minor losses, and may be correlated with Reynolds number (Re) [30]. Therefore, the correlation between a minor loss coefficient (K) associated with PEEP and Reynolds number was evaluated. Re was calculated using the characteristic air speed through the non-occluded nares area (*U*), determined by the flow rate (*Q*) divided by the area between the nostril walls and the outer wall of the cannula prongs (*A*_*non-occluded*_):

1 $$ U = \frac{Q}{{A_{{non - occluded}} }} $$

The hydraulic diameter (*D*_*h*_) was calculated by treating the area between the nostril walls and the outer wall of the cannula prongs as an annular cross-section:

2 $$D_{h}  = D_{{OD}}-D_{{ID}}$$

where the inner diameter of the nostril wall is *D*_*OD*_ and the outer diameter of the prong is *D*_*ID*_. With these definitions of *U* and *D*_*h*_, Re was:

3 $$Re = \frac{{\rho UD_{h} }}{\mu }$$

where density of air (ρ) at 34 °C was 1.15 kg/m^3^ and dynamic viscosity (*μ*) was 1.89E−5 kg/m*s.

A minor loss coefficient associated with PEEP was then calculated as:

4 $$ {{K}} = \frac{{2\left( {{{PEEP}}} \right)}}{{\uprho {{U}}^{2} }}  $$

### Continuous positive airway pressure

CPAP was delivered using a CPAP machine (S8 Elite; ResMed, San Diego, CA, USA) connected to a nasal mask (Infant Pocket Mask; nSpire Health Inc., CO, USA) through supply tubing including an exhalation port (Wisp tube and elbow assembly; Philips Respironics, Murrysville, PA, USA). Masks were sealed to the face of each child replica using silicone adhesive. A Pitot tube flow sensor (RespEQ, Baltimore, MD, USA) [31] was attached inline between the CPAP machine and the mask to measure the air flow in real time in standard litres per minute (SLPM; with standard conditions defined as 21.1 °C and 101.3 kPa). SLPM was converted to L/min during analysis using average conditions of the lab during the testing period (21.1 °C and 94.3 kPa; Edmonton, Alberta, Canada). The flow waveform was used to calculate the leak flow through the exhalation port, averaged over the breathing cycle, and to ensure that unintended mask leak was at a minimum. This mask leak measurement system was validated and used in a previous study by Duong et al. [16]. Two CPAP settings were selected for testing: 5 cmH_2_O and 10 cmH_2_O. These settings coincide with typical settings used for children of this age range [20].

### Study design

The study was done in two parts, one for assessing tracheal pressures and one for assessing EtCO_2_.

For tracheal pressures, CPAP settings of 5 and 10 cm H_2_O were tested for all 10 replicas. For NHF, the Optiflow Junior 2 nasal cannula was tested in all 10 replicas, but the Optiflow 3S and Optiflow + nasal cannulas were only tested in five replicas (subjects 3, 5, 11, 12, and 13), as prong sizes were too large to fit the nostrils of the other five replicas. A single test ran for approximately 30 breaths while tracheal pressures were recorded by the lung simulator. The pressures were each averaged over five breaths, breaths 21–25, and were used for further analysis. Each intervention was tested three times for each replica, and the NHF cannula prongs were repositioned between repetitions.

For EtCO_2_, three CPAP settings were tested for all 10 replicas: 5 cmH_2_O, 10 cmH_2_O, and zero CPAP (with the sealed mask in place). For NHF, similar to the pressure tests, the Optiflow Junior 2 was tested for all 10 replicas, but the Optiflow 3S and Optiflow + were tested for five replicas. A single test ran until EtCO_2_ reached steady state and was recorded, typically taking ~ 80 to 100 breaths. Again, each intervention was tested three times for each replica, and the NHF cannula prongs were repositioned between repetitions.

### Statistical analysis

A set of one factor repeated measures Analysis of Variance (ANOVA) procedures were done along with Tukey post hoc analysis comparing the tracheal pressures and change in EtCO_2_ between CPAP and NHF (n = 10). Three interventions were compared for the four tracheal pressure parameters: 5 cmH_2_O CPAP, 10 cmH_2_O CPAP, and the Optiflow Junior 2. Four interventions were compared for change in EtCO_2_: zero CPAP (sealed mask), 5 cmH_2_O CPAP, 10 cmH_2_O CPAP, and the Optiflow Junior 2. Results with two-sided *P* ≤ 0.05 was considered significant.

Another set of one factor repeated measures Analysis of Variance (ANOVA) procedures were done along with Tukey post hoc analysis comparing the tracheal pressures and change in EtCO_2_ between the three NHF cannulas (n = 5). Three interventions were compared for the four tracheal pressure parameters and change in EtCO_2_: the Optiflow 3S, the Optiflow +, and the Optiflow Junior 2. Results with two-sided *P* ≤ 0.05 were considered significant. Statistical analysis was performed with MATLAB (MathWorks, Natick, MA, USA). Tabulated results of all statistical tests performed are available as Additional file 1.

## Results

### Comparison of CPAP vs NHF

The delivered flow rate of air during CPAP, averaged over the breath, was measured as 18.8 ± 1.1 L/min for 5 cmH_2_O and 26.1 ± 1.6 L/min for 10 cmH_2_O (mean ± standard deviation; n = 10 replicas).

**Fig. 5:**
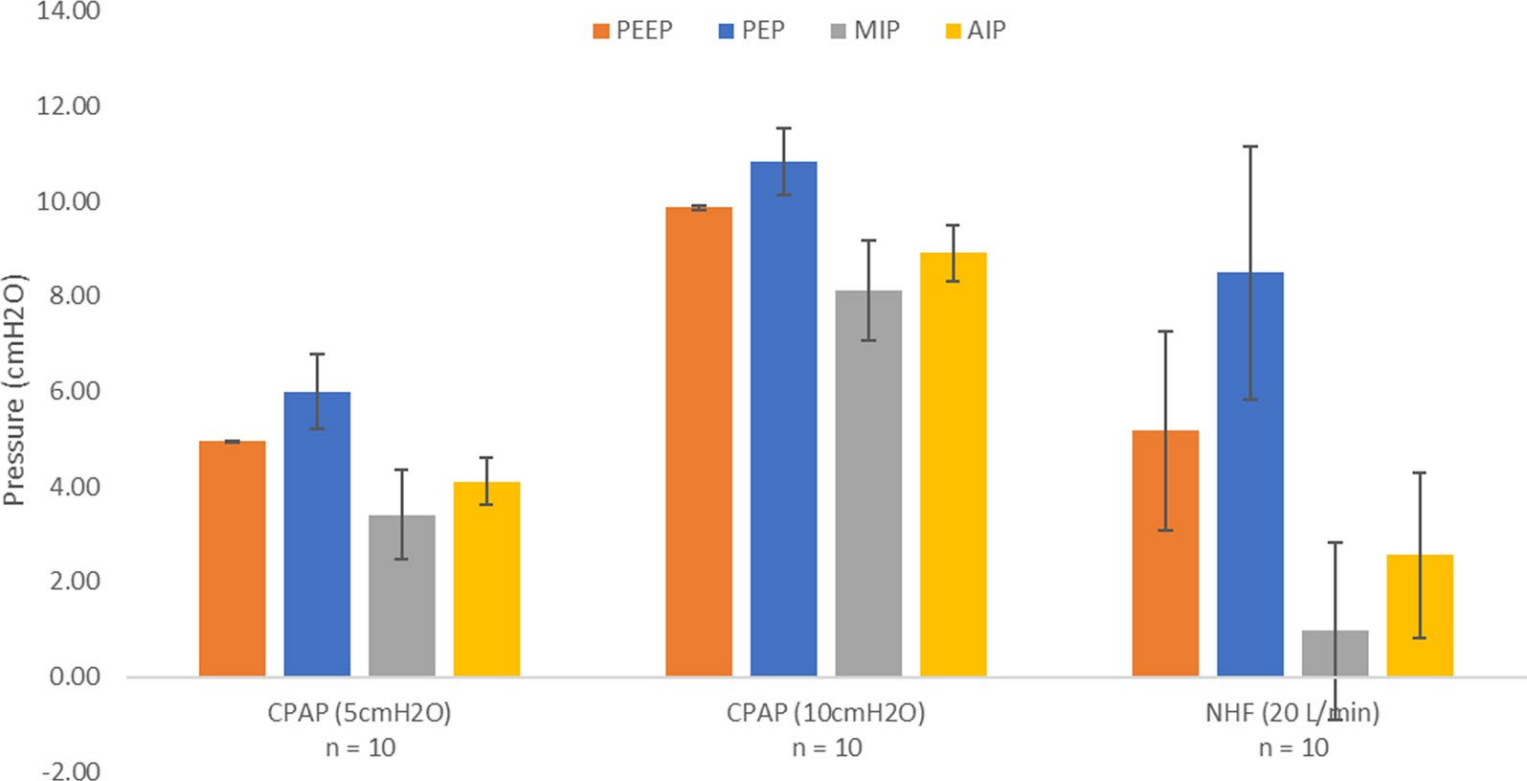
Average tracheal pressures across all 10 airway replicas for CPAP at 5cmH_2_O, CPAP at 10cmH_2_O, and NHF at 20 L/min (Optiflow Junior 2 cannula). Error bars represent one standard deviation around the average. *PEEP* positive end-expiratory pressure; *PEP* peak expiratory pressure; *MIP* minimum inspiratory pressure; *AIP* average inspiratory pressure

**Fig. 6:**
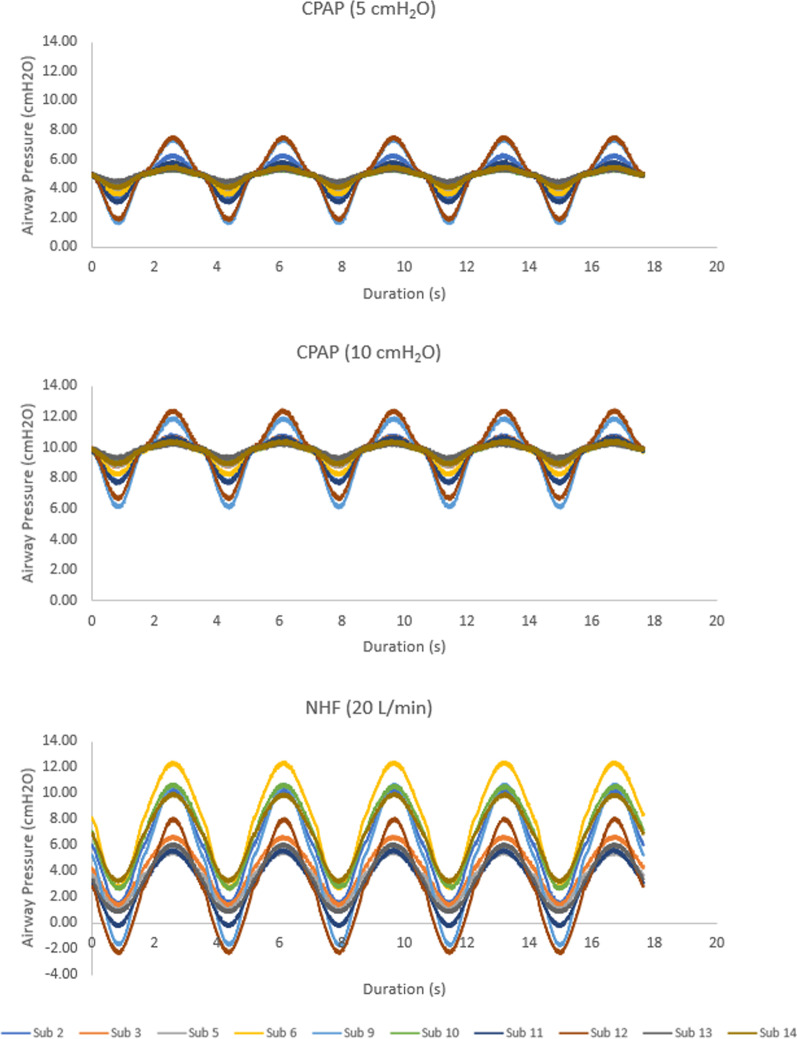
Tracheal pressure waveforms measured over 5 breaths during administration of 5 cmH_2_O CPAP (top), 10 cmH_2_O CPAP (middle), and NHF at 20 L/min (Optiflow Junior 2 cannula; bottom)

Average PEEP, PEP, MIP, and AIP across the 10 replicas for the three intervention types are displayed in Fig. 5. From ANOVA, the selection between CPAP and NHF was observed to have a significant influence on tracheal pressures. From post hoc analysis, 5 cmH_2_O CPAP was different from 10 cmH_2_O CPAP for all four pressure parameters, but different from NHF only in terms of PEP and MIP. 10 cmH_2_O CPAP was different from NHF in terms of PEEP, MIP, and AIP. Sample pressure waveforms for all individual replicas during administration of CPAP and NHF are displayed in Fig. 6.

**Fig. 7:**
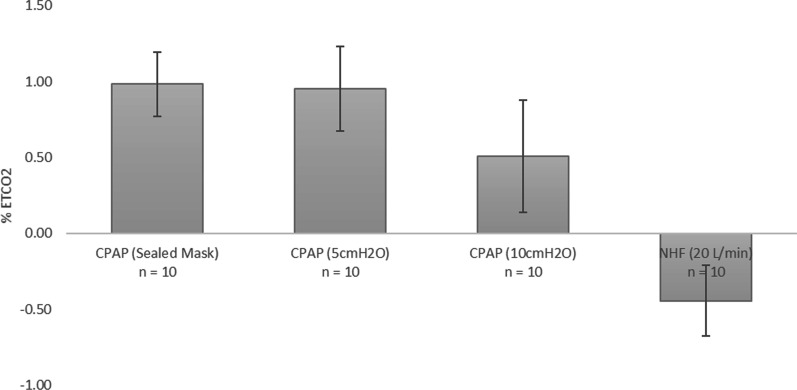
Average change in %EtCO_2_ from baseline across all 10 airway replicas for CPAP with sealed mask on (but zero CPAP applied), CPAP at 5cmH_2_O, CPAP at 10cmH_2_O, and NHF at 20 L/min (Optiflow Junior 2 cannula). Error bars represent one standard deviation around the average

Average change in EtCO_2_ from baseline across the 10 replicas for the four intervention types are displayed in Fig. 7. Selection between CPAP and NHF was observed to have a significant influence on change in EtCO_2_. From post hoc analysis, all interventions tested were different from one another in terms of average change in EtCO_2_, except for the pairing of zero CPAP (with the sealed mask in place) and 5 cmH_2_O CPAP.

### Comparison between three NHF cannulas

**Fig. 8:**
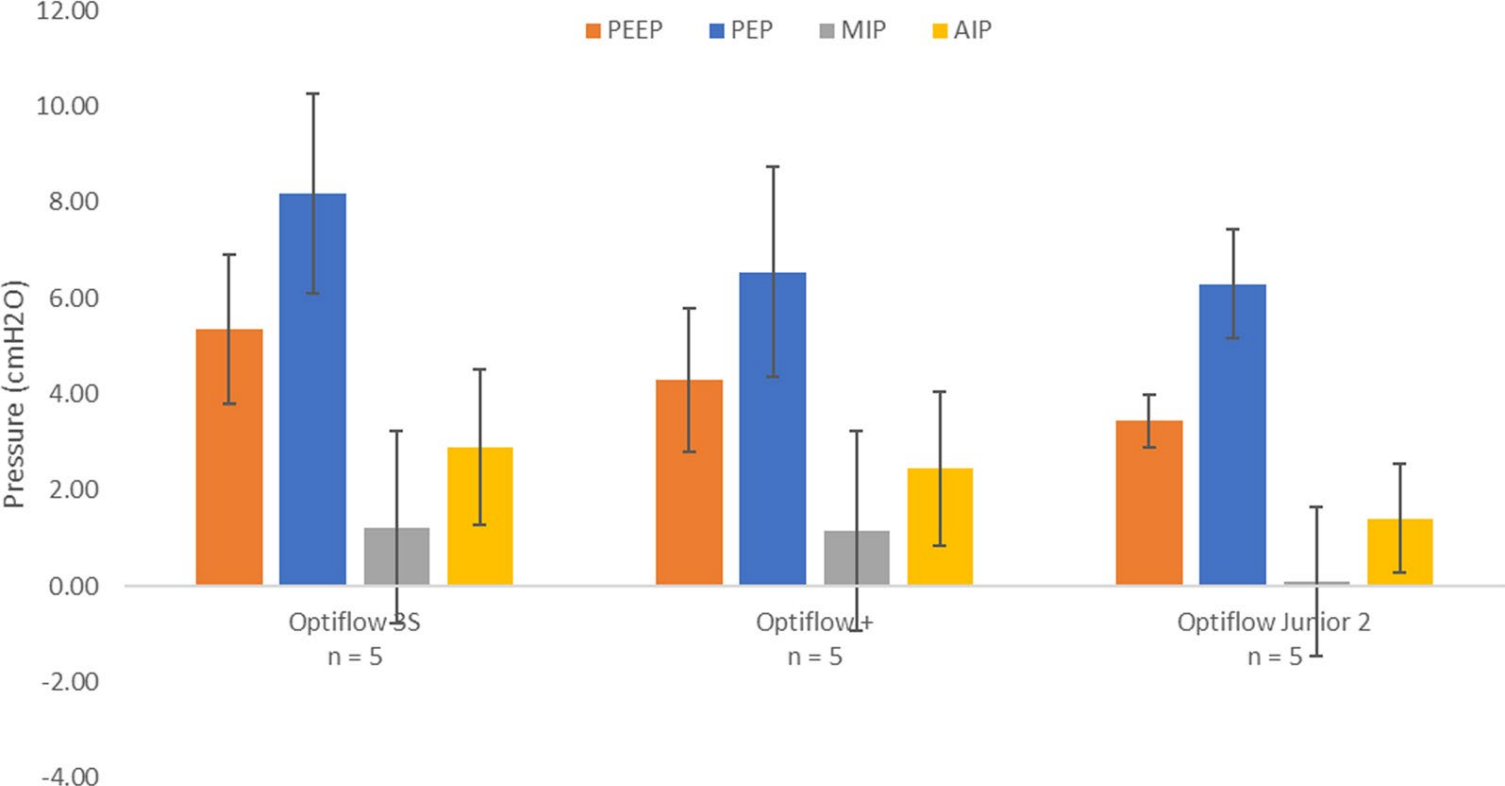
Average tracheal pressures across 5 airway replicas for three NHF cannulas, Optiflow 3S, Optiflow +, and Optiflow Junior 2. Error bars represent one standard deviation around the average. *PEEP* positive end-expiratory pressure; *PEP* peak expiratory pressure; *MIP* minimum inspiratory pressure; *AIP* average inspiratory pressure

**Fig. 9:**
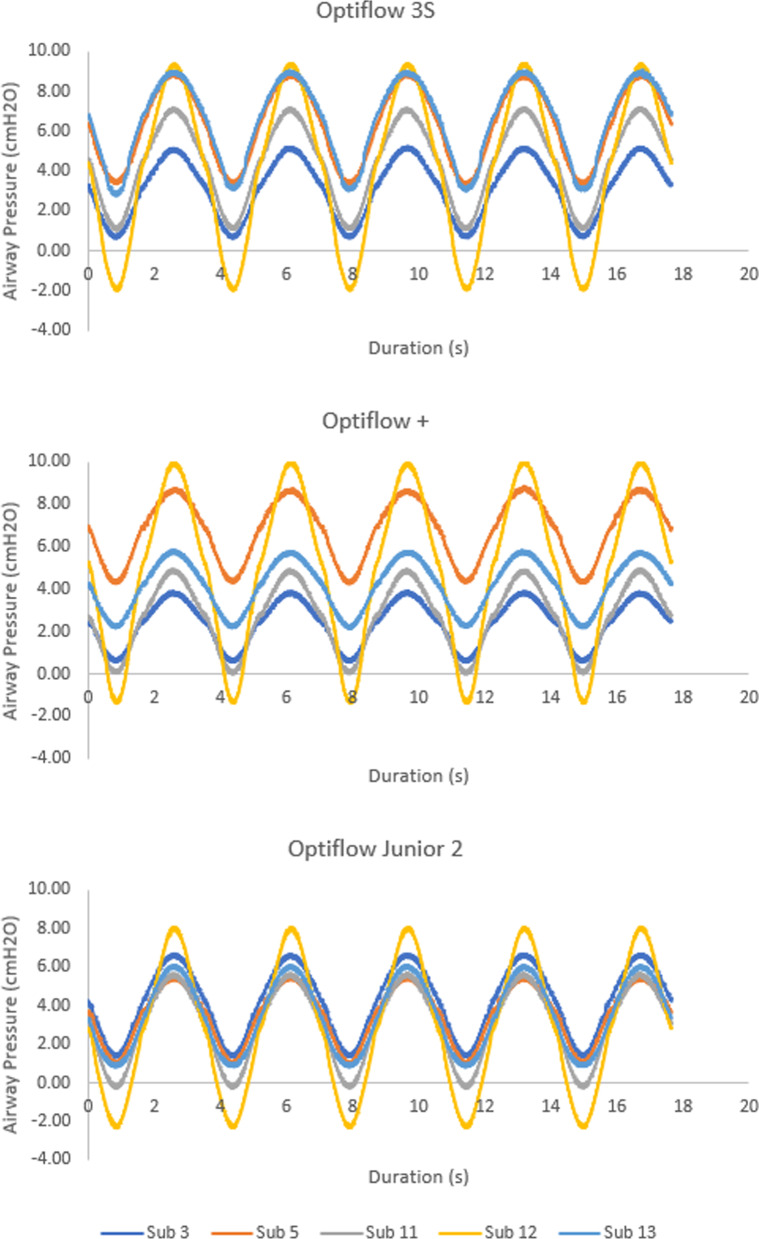
Tracheal pressure waveforms measured over 5 breaths during administration of NHF using the Optiflow 3S cannula (top), the Optiflow + cannula (middle), and the Optiflow Junior 2 cannula (bottom)

Average PEEP, PEP, MIP, and AIP across the five replicas tested with three different NHF cannulas are displayed in Fig. 8. From ANOVA, the selection of nasal cannula was not observed to have a statistically significant influence on tracheal pressures. Sample pressure waveforms for the five tested replicas during administration of NHF for all three nasal cannulas are displayed in Fig. 9.

**Fig. 10:**
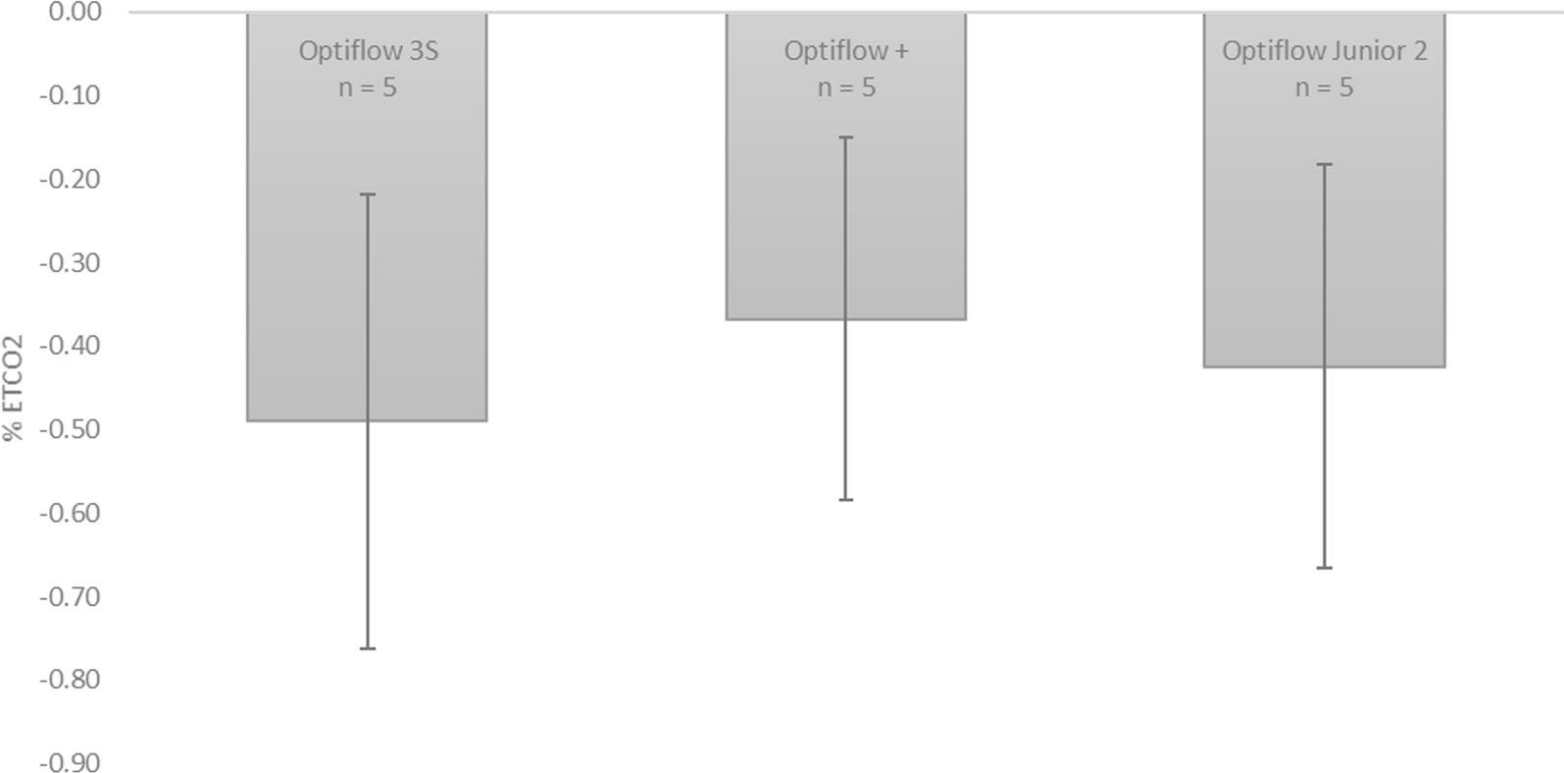
Average change in %EtCO_2_ from baseline across 5 airway replicas for the three NHF cannulas. Error bars represent one standard deviation around the average

Average change in EtCO_2_ from baseline across the five tested replicas for NHF are displayed in Fig. 10. Similar to tracheal pressures, selection of nasal cannula was not observed to have a statistically significant influence on change in EtCO_2_.

### Minor loss coefficients and Reynolds numbers

Across the three NHF cannulas and ten replicas, the Reynolds numbers calculated using Eq. 3 ranged from 950 to 1350. Minor loss coefficients calculated using Eq. 4 for the Optiflow 3S and Optiflow + cannulas, and averaged over five replicas, were 23 ± 4 and 20 ± 5, respectively (average ± standard deviation). The minor loss coefficient for the Optiflow Junior 2 cannula, averaged over the larger set of ten replicas, was 23 ± 13.

## Discussion

Results of in vitro experiments evaluating tracheal pressures and EtCO_2_ during delivery of CPAP or NHF to child airway replicas are reported above. Several differences between CPAP and NHF warrant further discussion, as do the potential sources of variability in pressure and gas washout between airway replicas.

For the delivery of CPAP, PEEP was observed to be approximately constant across the 10 airway replicas at either 5 cmH_2_O or 10 cmH_2_O (Fig. 5), indicating that the CPAP machine was working as intended, and delivered targeted positive airway pressures. In contrast, PEP, MIP, and AIP were observed to vary between replicas, indicating that these three pressure parameters were influenced by additional factors including breathing flow rates and the airway geometries of each subject (Figs. 5 and 6). This was expected, as airway pressure was evaluated at the exit of each replica (representative of a tracheal pressure), such that pressure drop through the replica influenced the airway pressure in all cases where flow was nonzero. In contrast, PEEP was measured at a point on the breathing cycle of zero flow, such that the instantaneous pressure drop through the replica is also zero.

Unlike the CPAP machine, the NHF system does not adjust delivered flow rate to maintain a constant pressure. As such, all pressure parameters, including PEEP, were observed to be variable across the 10 airway replicas for the delivery of NHF, with negative pressures observed during inhalation for 3 of 10 replicas (Figs. 5 and 6). With a set flow rate of 20 L/min, the average PEEP across the 10 airway replicas was approximately 5 cmH_2_O, which is similar to a CPAP setting of 5 cmH_2_O. Accordingly, though NHF can generate positive airway pressures, the pressures are variable and subject-dependent. McGinley et al. [20] reported on the delivery of NHF as an alterative to CPAP for children aged 10 ± 1 years (mean ± SEM; n = 12) at a set flow rate of 20 L/min. In their study, they found similar reductions in apnea–hypopnea index, comparable to CPAP prior to the study, when using NHF in a majority of the children studied [20]. Prior to NHF, the average CPAP setting used for therapy was 9 ± 1 cmH_2_O (mean ± SEM; n = 10) [20].

An increase in EtCO_2_ from baseline was observed during CPAP therapy across all 10 upper airway replicas. The presence of a mask increased EtCO_2_, due to added dead space of the mask. This increase was smallest for CPAP at 10 cmH_2_O (Fig. 7), owing to the greater average flow rate delivered from the CPAP machine at the higher CPAP setting. In contrast, a reduction in EtCO_2_ from baseline was observed during NHF therapy across all 10 upper airway replicas. This is consistent with a known mechanism of NHF: washout of the nasopharyngeal dead space, leading to reduced rebreathing of expired air [24, 32]. It is notable that, due to differences between the NHF cannula interface and CPAP mask interface, effective washout was observed for NHF at a flow rate of 20 L/min, whereas no, or limited, washout was observed for CPAP with an average delivered flow rate of 18.8 L/min (for CPAP at 5 cmH_2_O), or 26.1 L/min (10 cmH_2_O). During exhalation, any flow delivered by the CPAP machine is diverted through the exhalation port, such that little mixing occurs with gases in the mask or upper airway.

No significant difference was observed in tracheal pressures nor change in EtCO_2_ between the three different NHF cannulas for the subset of five tested replicas. An average PEEP of 5.4 ± 1.6 cmH_2_O, 4.3 ± 1.5 cmH_2_O, and 3.5 ± 0.5 cmH_2_O were generated through the Optiflow 3S, +, and Junior 2 nasal cannula, respectively (Fig. 8). Though not statistically significant, differences in average PEEP between cannula models may be associated with different cannula prong sizes, as has been noted to influence PEEP in previous studies [33, 34]. All three nasal cannulas also had similar reductions in EtCO_2_ from baseline: − 0.5 ± 0.3% for the Optiflow 3S, − 0.4 ± 0.2% for the Optiflow +, and − 0.4 ± 0.2% for the Optiflow Junior 2 (Fig. 10). However, only five replicas were tested because two of the three nasal cannula models, the Optiflow 3S and the Optiflow +, did not fit the five remaining replicas. This indicates that the selection of nasal cannula for NHF is important for fit and preventing blockage of the nares during delivery of therapy. Relationships between reduction in EtCO_2_ from baseline with tidal volume and replica volume were also investigated; however, no correlation was observed. It may be that variability in gas washout during NHF was influenced by the shape of the replica airways, especially the nasal vestibule in immediate proximity of cannula prongs; however, this was not investigated in detail in the present study.

The increased variability between replicas in tracheal pressures generated during NHF as compared to CPAP is noticeable in Figs. 5 and 6. Variability in PEEP between replicas was accounted for in part by modeling the pressure drop through the annular space between the prongs and nostril walls as a minor loss. Such a model is frequently adopted in fluid mechanics to calculate the pressure drop associated with flow through a constriction or past an obstruction. On average, calculated minor loss coefficients did not vary appreciably between the three NHF cannulas studied. Furthermore, minor loss coefficients remained approximately constant across the range of Reynolds numbers studied (Re = 950–1350), as is typically observed for flow through a constriction [30]. Similarly, Katz et al. [35] previously adopted a minor loss model for the pressure drop through extrathoracic and bronchial airways, and observed that minor loss coefficients approached constant values as Reynolds numbers exceeded ~ 1000. In the present work, this relationship suggests that PEEP generated in the replicas by NHF was related primarily to the occlusion of the nares by the cannula prongs. For a fixed flow rate of gas supplied to the cannula, the greater the extent of occlusion, the larger the PEEP that will be generated [36].

Some variability in calculated minor loss coefficients persisted between replicas, and can be attributed primarily to the dissimilar shape of the annular space for different replicas, which is not fully accounted for in the use of a single length scale, namely the hydraulic diameter calculated in Eq. 2. Variation in the percentage of the nostrils’ inlet area occluded by cannula prongs may also have contributed to variability between replicas in the minor loss coefficients. The greater variability in minor loss coefficient between replicas for the Optiflow Junior 2 cannula, as compared with the other two NHF cannulas studied, likely resulted from the larger number of replicas investigated with this cannula. For the subset of five replicas tested with all three NHF cannulas, the percent of occlusion ranged from 34 to 47% for the Optiflow 3S, 33–45% for the Optiflow +, and 20–27% for the Optiflow Junior 2. When tested over the larger set of 10 replicas, the percent of occlusion ranged from 20 to 41% for the Optiflow Junior 2.

Previously, Moore et al. [33, 34] identified predictive correlations for PEEP generated during application of NHF based on a characteristic air speed through the non-occluded nares area, as in Eq. 1 of the present study, but also influenced by an additional characteristic air speed exiting the cannula prongs. In the present work, consideration of this additional characteristic air speed did not further improve our ability to account for variability in PEEP between nasal cannulas. This may in part be due to the limited range of air speeds exiting cannula prongs in the present study, which was conducted with a single flow rate supplied to nasal cannula. Furthermore, the Moore et al. studies included high flow nasal cannula from a different manufacturer, which are intentionally designed with smaller inner diameters to influence washout of the upper airway [37].

A limitation of this study is the use of rigid airway replicas. They did not deform during breathing or under positive airway pressures, and thus the dynamic effects of breathing are not fully captured. Additionally, airway replicas used in the present study were fabricated based on scans of children that were obtained for indications other than airway pathology, whereas children with OSA may have reduced upper airway dimensions compared to controls [38]. We tried to minimize these limitations by testing multiple airway replicas to cover a range of differing airway geometries. Variation in, e.g., airway volume or cross-sectional areas between different airway replicas is expected to be much greater than variation that occurs dynamically over an individual’s breathing cycle. Furthermore, the range of airway dimensions measured in children with OSA overlaps that measured in controls [38], such that we expect the conclusions of the present work to extend to airway geometries representative of children with OSA. A second limitation is the testing of only one flow rate setting for NHF, 20 L/min, for our airway replicas with a subject age range of 4–8 years old. Previous studies have shown both airway pressures and washout to be flow rate dependent [33, 39]. However, clinical studies by McGinley et al. and Amaddeo et al. both used 20 L/min when investigating the use of NHF therapy as a treatment for OSA in children, aged 10 ± 1 years and 8.9 ± 6.2 years respectively [20, 21]. In both studies, NHF therapy at 20 L/min had a positive effect in treating OSA [20, 21]. Therefore, we focused on NHF at 20 L/min as a clinically-relevant flow rate for children with OSA.

## Conclusions

NHF delivered at 20 L/min to 4–8 year old child airway replicas generated average PEEP similar to CPAP at 5 cmH_2_O. Variation in PEEP, and in the maximum and minimum airway pressures recorded over the breathing cycle, was greater between airway replicas for NHF than for CPAP. Application of NHF reduced EtCO_2_ from baseline values, whereas delivery of CPAP through a sealed nasal mask increased EtCO_2_ from baseline values. NHF may benefit children who are non-compliant to CPAP therapy. Thus, further studies investigating NHF therapy as an alternative to CPAP therapy for treating OSA are warranted. These studies should consider potential beneficial effects of improved gas washout when administering NHF distinctly from the use of NHF to produce positive airway pressure.

## Supplementary Information

**Additional file 1.** Tabulated statistical results.

## Data Availability

The datasets used and/or analysed during the current study are available from the corresponding author on reasonable request.

## Abbreviations

AIP: Average inspiratory pressure
ANOVA: Analysis of variance
CPAP: Continuous positive airway pressure
EtCO_2_: End-tidal carbon dioxide
MIP: Minimum inspiratory pressure
NHF: Nasal high flow
OSA: Obstructive sleep apnea
PEP: Peak expiratory pressure
PEEP: Positive end-expiratory pressure
SLPM: Standard litres per minute
