# Supplementary material for: Comparison of airway pressures and expired gas washout for nasal high flow versus CPAP in child airway replicas
Source: Respir Res. 2021 Nov 10;22:289. doi: 10.1186/s12931-021-01880-z (PMC8579677; doi:10.1186/s12931-021-01880-z)
Supplement: Supplementary file 1 — Additional file 1. Tabulated statistical results. [file 12931_2021_1880_MOESM1_ESM.pdf]

Comparison of Airway Pressures and Expired Gas Washout for Nasal High Flow versus CPAP in Child Airway Replicas

Kelvin Duong[1], Michelle Noga[2], Joanna E. MacLean[3], Warren H. Finlay[1], Andrew R. Martin[1]

1. Department of Mechanical Engineering, University of Alberta
2. Department of Radiology and Diagnostic Imaging, University of Alberta
3. Department of Pediatrics and Women & Children's Health Research Institute, Faculty of Medicine & Dentistry, University of Alberta; Stollery Children's Hospital

Corresponding Author: Andrew Martin, 780-492-9012, andrew.martin@ualberta.ca, 10-324

Innovation Centre for Engineering, University of Alberta, Edmonton, Alberta, T6G 1H9, Canada

Additional Material: Tabulated Statistical Results

1). Comparison of Average Tracheal Pressures between CPAP (5cmH_2O and 10cmH_2O) and NHF (Optiflow Junior 2 at 20 L/min)

Average PEEP (CPAP vs NHF; n = 10)		
ANOVA		
P-Value	0.0000000355	
Post Hoc		
Pairs		P-Value
CPAP (5cmH2O)	CPAP (10cmH2O)	0.0000000132
CPAP (5cmH2O)	NHF (20 L/min)	0.938
CPAP (10cmH2O)	NHF (20 L/min)	0.000172

Average PPeak (CPAP vs NHF; n = 10)

ANOVA		
P-Value	0.00000650	
Post Hoc		
Pairs		P-Value
CPAP (5cmH2O)	CPAP (10cmH2O)	0.0000000132
CPAP (5cmH2O)	NHF (20 L/min)	0.0357
CPAP (10cmH2O)	NHF (20 L/min)	0.0521

Average Pmin (CPAP vs NHF; n = 10)

ANOVA		
P-Value	0.0000000000000655	
Post Hoc		
Pairs		P-Value
CPAP (5cmH2O)	CPAP (10cmH2O)	0.0000000132
CPAP (5cmH2O)	NHF (20 L/min)	0.000386
CPAP (10cmH2O)	NHF (20 L/min)	0.0000000715

Average Pinsp (CPAP vs NHF; n = 10)

ANOVA		
P-Value	0.00000000000936	
Post Hoc		
Pairs		P-Value
CPAP (5cmH2O)	CPAP (10cmH2O)	0.0000000132
CPAP (5cmH2O)	NHF (20 L/min)	0.0239
CPAP (10cmH2O)	NHF (20 L/min)	0.000000974

2). Comparison of Average $EtCO_2$ between CPAP (Sealed Mask, 5cmH_2O, and 10cmH_2O) and NHF (Optiflow Junior 2 at 20 L/min)

Average EtCO2 (CPAP vs NHF; n = 10)

ANOVA		
P-Value	0.000000000000221	
Post Hoc		
Pairs		P-Value
CPAP (Sealed Mask)	CPAP (5cmH2O)	0.984
CPAP (Sealed Mask)	CPAP (10cmH2O)	0.0371
CPAP (Sealed Mask)	NHF (20 L/min)	0.00000129
CPAP (5cmH2O)	CPAP (10cmH2O)	0.000273
CPAP (5cmH2O)	NHF (20 L/min)	0.00000149
CPAP (10cmH2O)	NHF (20 L/min)	0.0000880

3). Comparison of Average Tracheal Pressures between NHF interfaces (Optiflow 3S, Optiflow +, and Optiflow Junior 2) at 20 L/min

Average PEEP (NHF; n = 5)

ANOVA		
P-Value	0.0811	
Post Hoc		
Pairs		P-Value
Optiflow 3S	Optiflow +	0.241
Optiflow 3S	Optiflow Junior 2	0.156
Optiflow +	Optiflow Junior 2	0.578

Average PPeak (NHF; n = 5)

ANOVA		
P-Value	0.0857	
Post Hoc		
Pairs		P-Value
Optiflow 3S	Optiflow +	0.0905
Optiflow 3S	Optiflow Junior 2	0.19
Optiflow +	Optiflow Junior 2	0.958

Average Pmin (NHF; n = 5)

ANOVA		
P-Value	0.0796	
Post Hoc		
Pairs		P-Value
Optiflow 3S	Optiflow +	0.969
Optiflow 3S	Optiflow Junior 2	0.187
Optiflow +	Optiflow Junior 2	0.276

Average Pinsp (NHF; n = 5)

ANOVA		
P-Value	0.0805	
Post Hoc		
Pairs		P-Value
Optiflow 3S	Optiflow +	0.591
Optiflow 3S	Optiflow Junior 2	0.158
Optiflow +	Optiflow Junior 2	0.341

4). Comparison of Average $EtCO_2$ between NHF interfaces (Optiflow 3S, Optiflow +, and Optiflow Junior 2) at 20 L/min

Average EtCO2 (NHF; n = 5)		
ANOVA		
P-Value	0.0685	
Post Hoc		
Pairs		P-Value
Optiflow 3S	Optiflow +	0.194
Optiflow 3S	Optiflow Junior 2	0.107
Optiflow +	Optiflow Junior 2	0.495
